# The holocentric chromosome microevolution: From phylogeographic patterns to genomic associations with environmental gradients

**DOI:** 10.1111/mec.17156

**Published:** 2023-10-05

**Authors:** José Ignacio Márquez‐Corro, Santiago Martín‐Bravo, José Luis Blanco‐Pastor, Modesto Luceño, Marcial Escudero

**Affiliations:** ^1^ Departamento de Biología Molecular e Ingeniería Bioquímica Universidad Pablo de Olavide Seville Spain; ^2^ Jodrell Laboratory, Department of Trait Diversity and Function Royal Botanic Gardens, Kew Richmond UK; ^3^ Departamento de Biología Vegetal y Ecología Universidad de Sevilla Seville Spain; ^4^ Departamento de Biología, IVAGRO Universidad de Cádiz, Campus de Excelencia Internacional Agroalimentario (CeiA3) Cádiz Spain

**Keywords:** *Carex*, chromosomal speciation, Cyperaceae, hybrid dysfunction, landscape genomics, suppression recombination

## Abstract

Geographic isolation and chromosome evolution are two of the major drivers of diversification in eukaryotes in general, and specifically, in plants. On one hand, range shifts induced by Pleistocene glacial oscillations deeply shaped the evolutionary trajectories of species in the Northern Hemisphere. On the other hand, karyotype variability within species or species complexes may have adaptive potential as different karyotypes may represent different recombination rates and linkage groups that may be associated with locally adapted genes or supergenes. Organisms with holocentric chromosomes are ideal to study the link between local adaptation and chromosome evolution, due to their high cytogenetic variability, especially when it seems to be related to environmental variation. Here, we integrate the study of the phylogeography, chromosomal evolution and ecological requirements of a plant species complex distributed in the Western Euro‐Mediterranean region (*Carex* gr. *laevigata*, Cyperaceae). We aim to clarify the relative influence of these factors on population differentiation and ultimately on speciation. We obtained a well‐resolved RADseq phylogeny that sheds light on the phylogeographic patterns of molecular and chromosome number variation, which are compatible with south‐to‐north postglacial migration. In addition, landscape genomics analyses identified candidate loci for local adaptation, and also strong significant associations between the karyotype and the environment. We conclude that karyotype distribution in *C.* gr. *laevigata* has been constrained by both range shift dynamics and local adaptation. Our study demonstrates that chromosome evolution may be responsible, at least partially, for microevolutionary patterns of population differentiation and adaptation in *Carex*.

## 1 INTRODUCTION

Chromosome evolution is one of the most important drivers of biodiversity (Ayala & Coluzzi, 2005; Butlin, 2005; Hoffmann & Rieseberg, 2008; Stebbins, 1950). Chromosomal rearrangements are often associated with speciation (Rieseberg, 2001), as they have the potential to reduce gene flow between diverging populations (Grant, 1963). In angiosperms, karyotypic diversity is significantly associated with both clade species richness and diversification rates, which suggests chromosomal rearrangements promote or reinforce the speciation process (Carta & Escudero, 2023). Much research into chromosome evolution in plants has focused on the physiological and ecological implications of polyploidy (e.g. Otto, 2007; Otto & Whitton, 2000), while the evolutionary consequences of dysploidy have received comparatively much less attention (Escudero et al., 2014; Mandáková & Lysak, 2018).

Speciation mediated by chromosome evolution has been explained by two models. First, the hybrid‐dysfunction model of chromosomal speciation presumes reduced fitness of hybrids between chromosome races (Ayala & Coluzzi, 2005). This model is undermined by the fact that strong selection against structural heterozygotes (underdominance) would make new cytotypes extremely difficult to succeed, meaning that underdominant mutations are not likely to cause speciation (Spirito, 1998). The alternative model, the suppressed recombination model of chromosomal speciation is better supported theoretically as it does not involve underdominance (Ayala & Coluzzi, 2005). In this case, chromosomal rearrangements promote divergence between populations by preventing recombination in clusters of locally coadapted gene complexes (supergenes; Schwander et al., 2014; Black & Shuker, 2019). These rearrangements may facilitate species divergence even in the presence of ongoing gene flow between chromosomatically differentiated populations (Lowry & Willis, 2010; Navarro & Barton, 2003; Noor et al., 2001; Ortíz‐Barrientos et al., 2002).

In holocentric chromosomes, centromeric regions are distributed along the entire length of the chromosome, which may therefore attach to microtubules during mitosis and meiosis (Márquez‐Corro, Martín‐Bravo, Pedrosa‐Harand, et al., 2019). In contrast, monocentric chromosomes have microtubule attachments localised exclusively in a single centromeric region. Holocentric chromosome organization has been described for ca. 20 lineages in three of the six kingdoms in the domain Eukarya (the Eukaryotes): plants (angiosperms and green algae); animals (at least six species‐rich arthropod clades, plus velvet worms and nematodes); and Rhizaria (Escudero et al., 2016; Márquez‐Corro et al., 2018). As a consequence, approximately 20% of all Eukaryotes have holocentric chromosomes (Márquez‐Corro et al., 2018). The evolutionary implications of holocentricity are potentially profound but largely elusive. Because of the special ability of holocentric chromosomes to create and maintain karyotype variation in the populations, they have been suggested as ideal systems to test the proposed models of chromosomal speciation (Lucek et al., 2022).

Chromosome fragments from fissions that would be lost in monocentric chromosomes may be inherited and become fixed in organisms with holocentric chromosomes (Márquez‐Corro, Martín‐Bravo, Pedrosa‐Harand, et al., 2019). Likewise, enlarged chromosomes from fusion events can align and segregate correctly in holocentric chromosomes. Conversely, in organisms with monocentric chromosomes fusions usually result in the formation of dicentric chromosomes that fail to segregate properly (Márquez‐Corro, Martín‐Bravo, Pedrosa‐Harand, et al., 2019). Consequently, large series of chromosome numbers and high rates of karyotype evolution are found in holocentric lineages (i.e. butterflies and moths—Lepidoptera—2*n* = 10 to 250, sedges—Cyperaceae—2*n* = 4 to 226; de Vos et al., 2020; Márquez‐Corro, Martín‐Bravo, Spalink, et al., 2019; Márquez‐Corro et al., 2021). In Lepidoptera, phylogenetic comparative evidence suggests that chromosome fission and fusion drive cladogenesis (Augustijnen et al., 2023; de Vos et al., 2020). In the sedge genus *Carex* (Cyperaceae), chromosome rearrangements contribute to genetic diversity within species (Escudero, Weber, et al., 2013; Hipp et al., 2010) and the karyotype diversity is positively associated with the time of coalescence of the species (Escudero et al., 2010). Finally, chromosome number changes from fission and fusion determine recombination rates as the number of chiasmata during meiosis is directly proportional to chromosome number (Nokkala et al., 2004). The reason for this is that there are interferences between segregation and recombination processes that are spatially separated in monocentric but not in holocentric chromosomes (Márquez‐Corro, Martín‐Bravo, Pedrosa‐Harand, et al., 2019). For example, in the holocentric *Carex scoparia*, recombination rates have been found to be proportional, on average, to only one chiasma per pair of homologous chromosomes during meiosis (Escudero et al., 2018). As a consequence, recombination rates (chromosome numbers) have been positively associated with adaptive speciation toward different environmental conditions in holocentric sedges (Escudero et al., 2012; Escudero, Maguilla, et al., 2013; Márquez‐Corro et al., 2021; Spalink et al., 2018). Some of the patterns of chromosome number association with environmental conditions are rather weak at the macroevolutionary level (Escudero et al., 2012; Márquez‐Corro et al., 2021; Spalink et al., 2018), probably based on the fact that chromosome evolution is much faster than speciation rates in sedges (Márquez‐Corro et al., 2021).

We hypothesise that holocentric chromosomes may be playing an important role in species' ability to adapt and colonise new environments. Therefore, having a direct impact on niche expansion for some of the most widespread species of the study group. Karyotype rearrangements may leave a traceable signature in the different individuals' genomes, probably linked to specific loci in the linkage groups. Landscape genomics constitutes a currently developing approach that may help to identify specific genetic markers, including chromosome number variation, associated with environmental variables and therefore involved in local adaptation processes (Ahrens et al., 2018; Hoban et al., 2016; Rellstab et al., 2015). To our knowledge, no study using landscape genomics has addressed this topic in organisms with holocentric chromosomes.

The goals of this study are: (i) to infer patterns of holocentric chromosome evolution at phylogeography scale in a lineage of four closely related and recently diversified *Carex* species, (ii) to identify genomic signatures of adaptive evolution and investigate the associations between chromosome numbers and local environmental conditions, (iii) to elucidate the role of cytogenetic evolution on the diversification of this plant lineage and (iv) to test the model of chromosomal speciation that could fit better this study group. Under a hybrid dysfunction model of chromosome speciation, we would expect clear genetic differences among different cytotypes that are not locally adapted (we do not expect a significant association between chromosome number and environmental variables under this model). Under a suppression of recombination model of speciation, we would not expect clear genetic differences among different cytotypes (different cytotypes do not necessarily show clear genetic differences) that are locally adapted to environmental conditions (we expect an association between chromosome number and environmental variables). Finally, under Lucek et al.'s (2022) framework for holocentric chromosome speciation both models may act synergistically during the speciation process: chromosome variation accumulates within species which may alter both recombination patterns and matting patterns, specially, between the most divergent karyotypes.

## 2 MATERIALS AND METHODS

### 2.1 Study group

**FIGURE 1:**
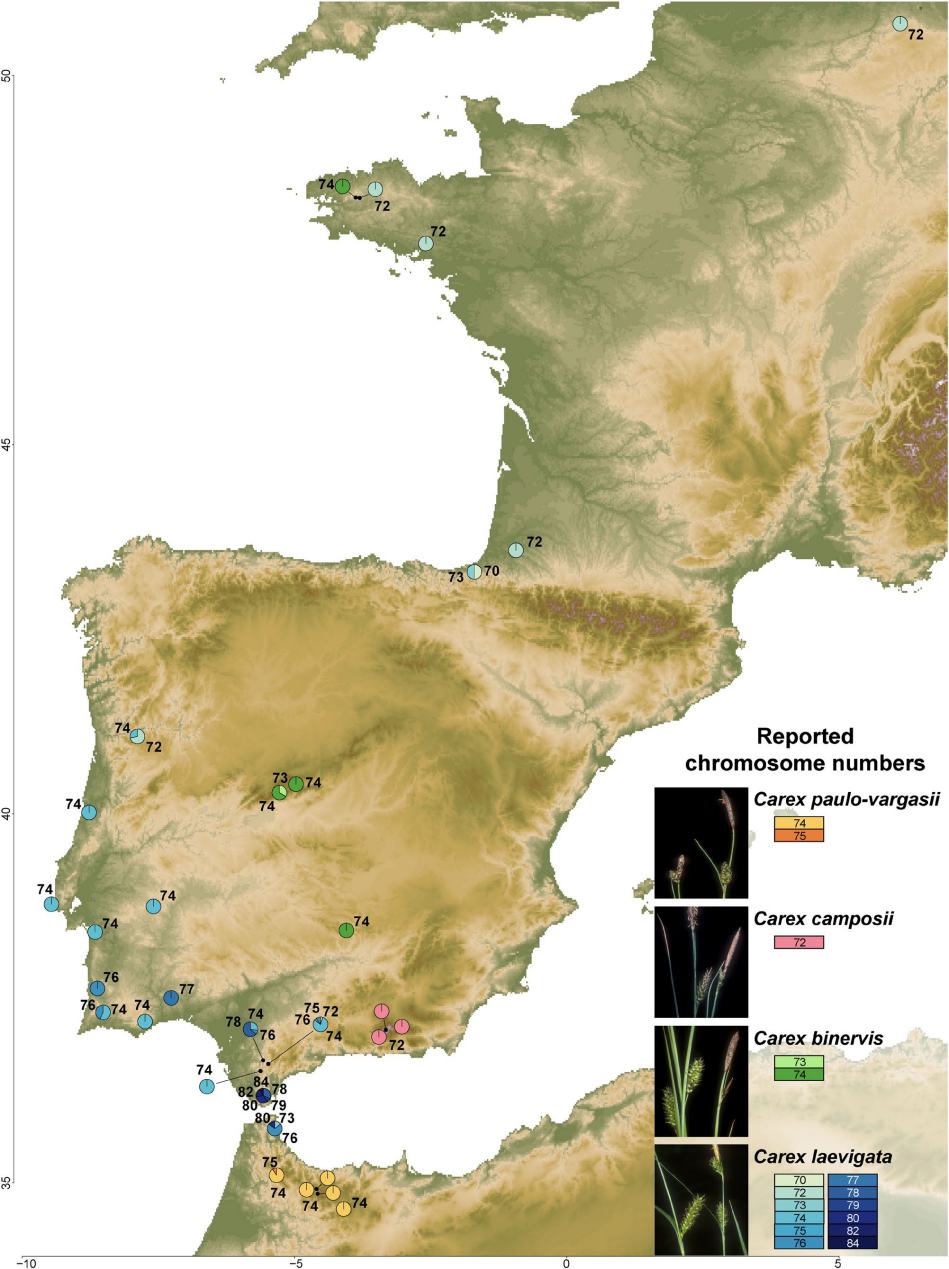
*Carex* group *laevigata* distribution of chromosome number for the populations sampled. Chromosome numbers (2*n*) for each population are indicated with colour‐coded pie charts displaying the relative proportion of sampled individuals with different chromosome numbers.

We selected a lineage within *Carex* sect. *Spirostachyae* (hereafter *C*. gr. *laevigata*) which is composed of four species (*C. laevigata*, *C. binervis*, *C. camposii* and *C. paulo‐vargasii*) endemic to western Mediterranean‐Atlantic Europe (Figure 1), due to several characteristics that make it ideal for the study of the possible association of chromosome number variation and specific genomic loci with environmental gradients. In the first place, their well‐known cytogenetic variability, with a representative sampling of chromosome counts across their relatively restricted distribution range (Luceño & Castroviejo, 1991). In the second place, their wide chromosome number variation at the inter‐, intraspecific (*C. binervis*, 2*n* = 72–74; *C. camposii*, 2*n* = 72; *C. paulo‐vargasii*, 2*n* = 74–75; *C. laevigata*, 2*n* = 69–84), and even intrapopulation level (specially in *C. laevigata*; Figure 1). Moreover, chromosome number variation in *C. laevigata* appears to be influenced by population latitude (Figure 1), which suggests a role of chromosome evolution in the adaptation to the environment (Escudero, Maguilla, et al., 2013; Luceño & Castroviejo, 1991). *Carex* gr. *laevigata* has a relatively recent origin (Pleistocene crown diversification; ca. 2 mya, Martín‐Bravo et al., 2019), and complex, previously unresolved evolutionary relationships, in which rapid chromosome speciation have been suggested to play an important role (Escudero, Maguilla, et al., 2013).

### 2.2 DNA extraction, sequencing and data clustering

A total of 189 individuals from 33 populations (from one to 28 individuals per population; Figure 1; Table S1) of the four species of *C*. gr. *laevigata* (plus one sample each of *C. mairei*—sister group—, *C. distans*, *C. boryana* and *C. borbonica* also from *Carex* section *Spirostachyae* as outgroup; Escudero & Luceño, 2009) were sampled. These individuals were selected because we had chromosome number information for 186 out of 189 individuals from previous studies (Escudero, Maguilla, et al., 2013). The sampling was carefully designed to account for the cytogenetic variation across species distributions. Thus, the sampling was more intense (more populations and individuals per population) in *C. laevigata*, the species with the highest inter‐ and intrapopulation chromosome variation and with the widest distribution (Escudero et al., 2013; Luceño & Castroviejo, 1991). On the other hand, we sampled less populations and individuals per population (typically one) for the species with reduced chromosome variation) and/or with a more restricted distribution (*C. binervis*, *C. paulo‐vargasii* and *C. camposii*; Escudero et al., 2010; Escudero, Maguilla, et al., 2013).

Restriction‐site‐associated DNA sequencing (RAD‐seq) has been proven as a useful high throughput sequencing approach for investigating recent evolutionary processes in non‐model organisms, including different scenarios of speciation among closely related species (Otero et al., 2022 and references therein), in which traditional Sanger‐sequenced nuclear and plastid markers have been unable to solve phylogenetic relationships (also in *C*. gr. *laevigata*; see Escudero, Maguilla, et al., 2013). A special effort was conducted for *C. laevigata* in order to obtain a wide intraspecific genomic sampling representative of chromosome number variation within and across populations. Thus, 133 individuals from 20 populations (minimum = 1 individual per population, 1st quartile = 2, median = 3.5, 3rd quartile = 7.25, maximum = 28) of *C. laevigata*, most of which with available chromosome counts (98.5% of the specimens; Table S1), were included. DNA was extracted using a DNeasy Plant Mini Kit (Qiagen, Valencia, CA, USA). RAD‐seq libraries preparation using restriction enzyme PstI were followed by sonication and barcoding performed by Floragenex (Eugene, Portland, OR, USA) following Baird et al. (2008).

Raw data were demultiplexed based on the code for each sample and adapters were removed from the reads using ipyrad v0.7.24 (Eaton & Overcast, 2020). We used the matrix resulting from a clustering threshold of 90% similarity and a minimum sequencing depth of 6 sequences per locus for within‐sample clustering (c90m6). We compared this matrix to others with a clustering of 95% and sequencing depth of 6 and 11, but no significant difference was found. Then we discarded those samples with higher missing data. The clusterings within and among samples were conducted using the reference genome option as implemented in ipyrad. By the time the analyses were made only three *Carex* genomes were available, two from subgenus *Vignea* (Planta et al., 2022) and one from subgenus *Euthyceras* (Can et al., 2020), that are equally related to subgenus *Carex* (Escudero, Marques, et al., 2023). We accordingly selected one of the genomes from subgenus *Vignea*, which genome assemblage was of the finest quality (*Carex scoparia*, section *Ovales*; Planta et al., 2022). Finally, we reanalyzed the data according to the c90m6 parameters and a minimum number of samples with data at a given locus of 90 out of 156 (ca. 60%). We discarded those samples with >95% of missing data for the phylogeny construction, so a final matrix of 156 accessions was retained (three outgroup taxa, one sister species and 152 *Carex* gr. *laevigata* samples). Accordingly, the final RADseq sampling included 152 *C*. gr. *laevigata* inviduals from 33 populations (minimum = 1 individual per population, 1st quartile = 2, median = 3, 3rd quartile = 5, maximum = 17). Only 16 populations were affected by the filters, with an average of ca. 30% reduction in the represented individuals, with only one population of *C. binervis* (B6) ‐with a single sampled individual—completely eliminated from the analysis (Table S2).

The phylogenetic relationships of the retained samples were reconstructed using IQ‐TREE v1.6.11 software (Hoang et al., 2018; Nguyen et al., 2015). The analysis was set to run 1000 Ultrafast Bootstrap approximation (UFBoot) with a hill‐climbing nearest‐neighbour interchange (NNI) search optimization and 1000 Shimodaira‐Hasegawa approximate likelihood ratio test (SH‐aLRT) replicates to assess branch support and the tree topology, using *C. borbonica* as outgroup.

#### 2.2.1 Genetic

In order to identify genetic structure within our sampling, we filtered the RADseq dataset further, retaining 117 individuals from *C*. gr. *laevigata* and in a matrix of 1337 loci (max. 17% of missing data per sample). Twenty‐three populations were affected by the filters, with an average of ca. 47% reduction in the represented individuals, and three populations were completely eliminated from the analysis (*C. binervis* B1 and B6 populations, and *C. laevigata* L5; Table S2). We used a Bayesian approach under an explicit population genetic model to find clusters of individuals under Hardy–Weinberg equilibrium and random mating and allowing admixture of populations and individuals. STRUCTURE v 2.3.2 (Pritchard et al., 2000) uses Markov chain Monte Carlo (MCMC) to recover a posterior probability (PP) distribution of population partitions and population genetic parameters using the admixture, correlated allele frequencies model. STRUCTURE simulations were run from *K* = 1 to 30 populations, with ten replicates per run of 100,000 iterations with a burn‐in of 10,000 iterations. The best‐fit value of K was estimated using the software STRUCTURE HARVESTER (Earl & VonHoldt, 2012), using delta K (Evanno et al., 2005) and taking into account the K selection concerns raised by Janes et al. (2017). The admixture graphic was generated using CLUMP AK (Kopelman et al., 2015).

### 2.3 Environment‐associated loci (RDA analysis)

We used redundancy analysis (RDA) to detect candidate genes under environmental selection in *C*. gr. *laevigata*. We performed four analyses with four different dataset: (i) 153 samples from 34 populations (*C*. gr. *laevigata* plus *C. mairei*), with 698 SNPs (see RADseq results) and chromosome number as an additional locus, (ii) 153 samples from 34 populations, with 698 SNPs and without chromosome number as an additional locus, and (iii) 53 samples from 34 populations, with 698 SNPs and chromosome number as an additional locus (each sample per population representing a different chromosome number when there is chromosome number variation within populations; when a chromosome number within a population is represented by 2 or more samples, one was randomly selected). RDA can be used as a genotype‐environment association (GEA) method to detect loci under selection based on multivariate ordination (Forester et al., 2016). RDA determines how groups of loci (here also chromosome number) covary in response to the multivariate environment and can detect processes that result in weak, multilocus molecular signatures (Forester et al., 2018; Rellstab et al., 2015). Compared to other methods, RDA has shown a superior combination of low false‐positive and high true‐positive rates across a variety of selection scenarios (Forester et al., 2018). Another advantage of RDA is that it can be used to analyse many loci and environmental predictors simultaneously. For RDA we considered only SNP sites for which there was sequence coverage in at least 100 individuals, and for which the less‐common allele was present in at least 10% of sampled individuals [minor allele frequency (MAF) filter ≥0.10]. The chromosome number was added as an additional column in the SNPs database. RDA is a regression‐based method, and so it is subject to problems when using highly correlated predictors. Hence, we removed correlated predictors with a correlation value of *r* > .7. We used bioclimatic variables from the Worldclim 2 database (Fick & Hijmans, 2017). The variable reduction was guided by an ecological interpretation of the relevance of possible predictors. We implemented a variable reduction protocol: First, we performed cluster analyses of factors according to a matrix of absolute correlation values |*r*|. For that, we used the complete linkage clustering method of the ‘hclust’ function in R (R Core Team, 2021). After subsequent cluster analyses, we retained one variable in clusters with a distance among variables lower than 0.3 (correlation higher than 0.7). We favoured quarterly over monthly variables.

We also checked for multicollinearity using variance inflation factors (VIF) and confirmed that VIF of selected variables was <10. We also performed a permutation test using 1000 permutations to assess the statistical significance of environmental variables and RDA axes used in the models. We used the loadings of the SNPs (stored as species in the RDA object from the R environment) in the ordination space to determine which SNPs are candidates for local adaptation. We extracted the SNP loadings from the two significant constrained axes. Outliers were identified as SNPs with the greatest loadings along the significant RDA axes (i.e. those in the 2.5% upper and lower tails; Capblancq et al., 2018), and were putatively considered as extremely associated with environmental variables. Then we investigated the correlations between environmental predictors and outlier SNPs. Finally, we represented SNPs in the ordination space and colour‐coded them based on the predictor variable with which they are most strongly correlated. Outlier SNPs with the same signal as chromosome number were selected for investigating their functional annotation, thus providing additional evidence of their role in environmental adaptation. We used their genomic position to identify candidate genes potentially under environmental selection located <50 Kbp upstream or downstream in the annotated *Carex scoparia* genome (Planta et al., 2022).

## 3 RESULTS

### 3.1 RAD‐seq output

After filtering the data through the ipyrad pipeline, we obtained two datasets: (i) one composed of 156 samples (*C*. gr. *laevigata* plus *C. mairei*, *C. distans*, *C. boryana* and *C. borbonica*), with 1761 loci and 10,138 SNPs filtered, that was subsequently used for phylogenetic analysis, (ii) one composed of 117 samples and 1337 SNPs filtered, that was subsequently used for STRUCTURE analysis (the maximum missing data allowed was 17%), and (iii) another retaining 153 samples (three outgroup samples excluded) with 698 SNPs filtered, that was used for RDA analyses.

### 3.2 Phylogenetic relationships

**FIGURE 2:**
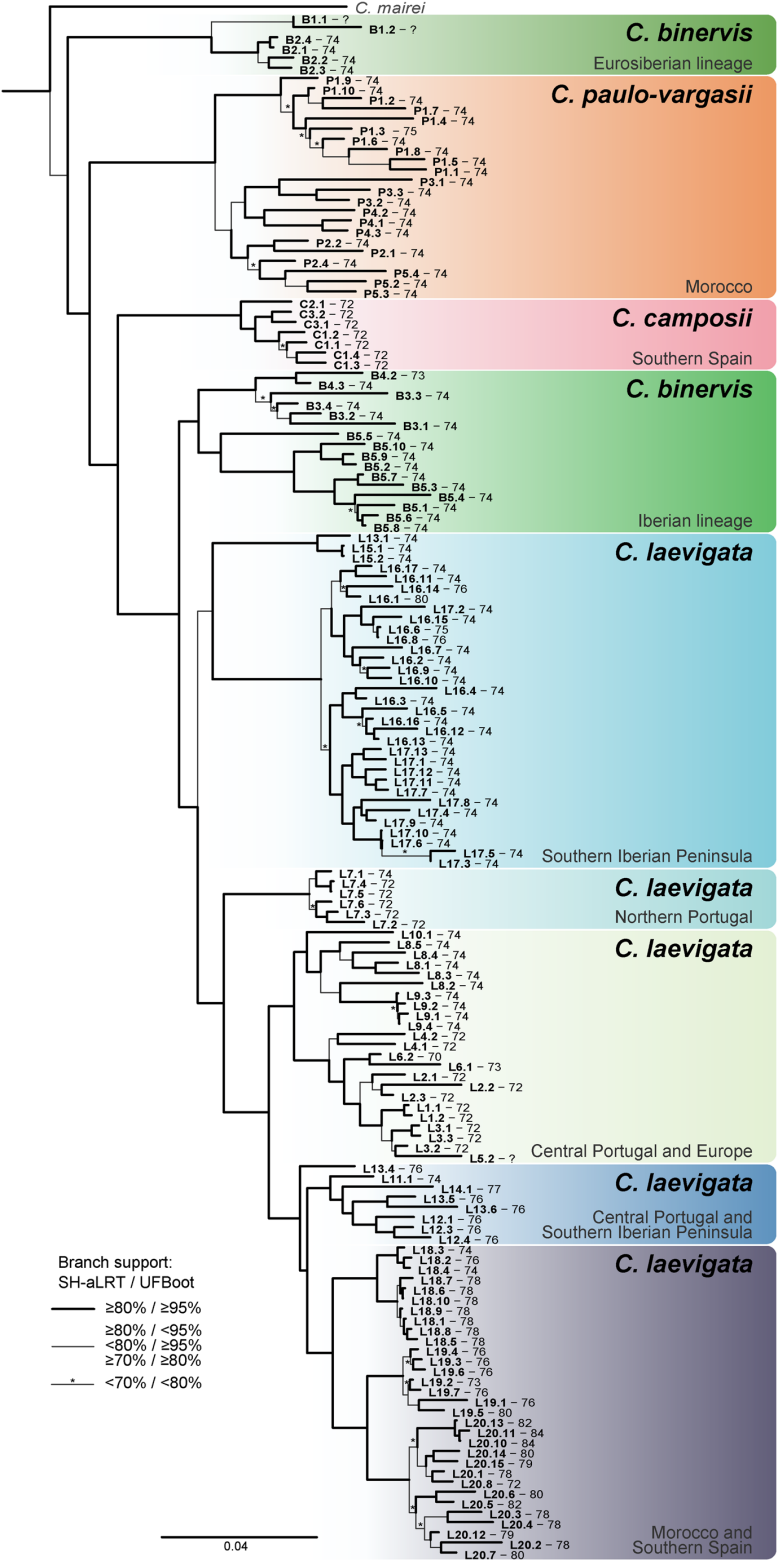
RAD‐seq phylogeny of 152 samples of *Carex* gr. *laevigata* and one sample of *C. mairei* (the three outgroup samples were dropped) based on 1761 loci and 10,138 SNPs filtered from IQ‐TREE analysis. The tip labels indicate species (B, P, C and L for *C. binervis*, *C. paulo‐vargasii*, *C. camposii* and *C. laevigata*, respectively), population number, individual number and—separated by a dash—diploid chromosome number. Distribution of the main lineages is specified next to the tree.

The phylogenetic relationships among *C*. gr. *laevigata* species established by the c90m6 matrix of RAD‐seq SNPs (Figure 2; Figure S1) are congruent with the previous studies but resolved the intermingled relationships that have been previously obtained for *C. laevigata‐C. binervis* (Escudero et al., 2008; Escudero & Luceño, 2009). *Carex paulo‐vargasii* and *C. camposii* were retrieved as subsequent sisters to the remaining species. *Carex laevigata* constitutes a monophyletic species, while *C. binervis* was retrieved as polyphyletic, with two distinct lineages, an early‐diverging one (sister to all *C*. gr. *laevigata*) that is composed of populations from France and the British Islands (Eurosiberian part of the species range), and an Iberian (Mediterranean part of the species range) *C. binervis* lineage that was retrieved as sister to *C. laevigata*. In turn, *C. laevigata* was composed of a main clade restricted to the southern Iberian Peninsula (2*n* = 72–76), sister to another main clade with a wider distribution, spanning the species range and chromosome number variability (2*n* = 69–84).

#### 3.2.1 Genetic structure analysis

**FIGURE 3:**
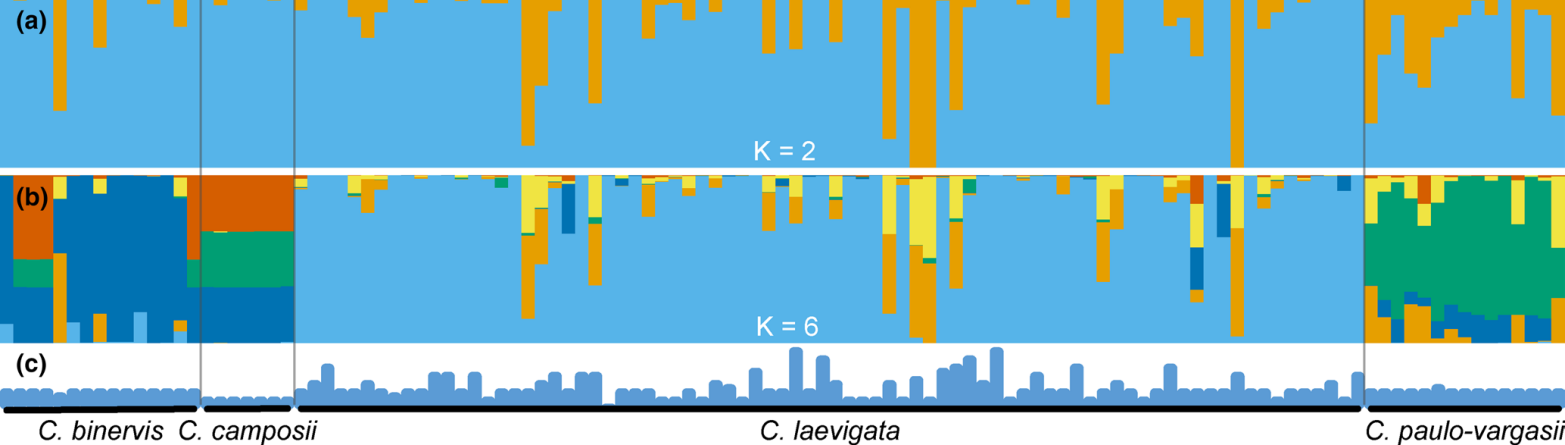
STRUCTURE analysis of *Carex* gr. *laevigata*. Results of the clustering for (a) *K* = 2 and (b) *K* = 6 values. (c) Chromosome number of the analysed individuals, ranging from 2*n* = 70 to 2*n* = 84 chromosomes.

The best STRUCTURE clusterings were *K* = 2 (delta*K* = 6477.4, Figure 3) and *K* = 6 (delta*K* = 75.9, Figure 3) followed by *K* = 3 (delta*K* = 6.1) and *K* = 13 (delta*K* = 4.6; Figures S2 and S3, Table S3). For *K* = 2, populations were grouped into: (i) cluster 1 in light blue across all samples and species but with near full clustering for Eurosiberian *C. binervis*, *C. camposii* and many individuals and populations from *C. laevigata*, (ii) cluster 2 in orange more frequent in *C. binervis* from the Iberian Peninsula, *C. paulo‐vargasii* and nine individuals from *C. laevigata* (seven from southern Spain, one from southern Portugal and one from France). For *K* = 6, populations were grouped into: (i) cluster 1 in light blue in most of the individuals from *C. laevigata*, (ii) cluster 2 in orange only with a small percentage in individuals from *C. binervis* from Iberian Peninsula (with one exception) and *C. paulo‐vargasii* and also a variable percentage of individuals from *C. laevigata*, most of them with a southern distribution, (iii) cluster 3 in dark blue in *C. binervis* from Iberian Peninsula (although a small percentage of this cluster can be found in *C. binervis* from Europe, *C. camposii* and *C. paulo‐vargasii* and exceptionally a few individuals from *C. laevigata*), (iv) cluster 4 in green, more frequent in *C. paulo‐vargasii* and also in *C. camposii* (also found in an smaller percentage in *C. binervis* from Europe), (v) cluster 5 in red more frequent in *C. binervis* from Europe and second in *C. camposii* and (vi) cluster 6 in yellow found in scattered individuals of *C. binervis* from Iberian Peninsula, *C. laevigata* and *C. paulo‐vargasii*.

### 3.3 RDA analysis

**FIGURE 4:**
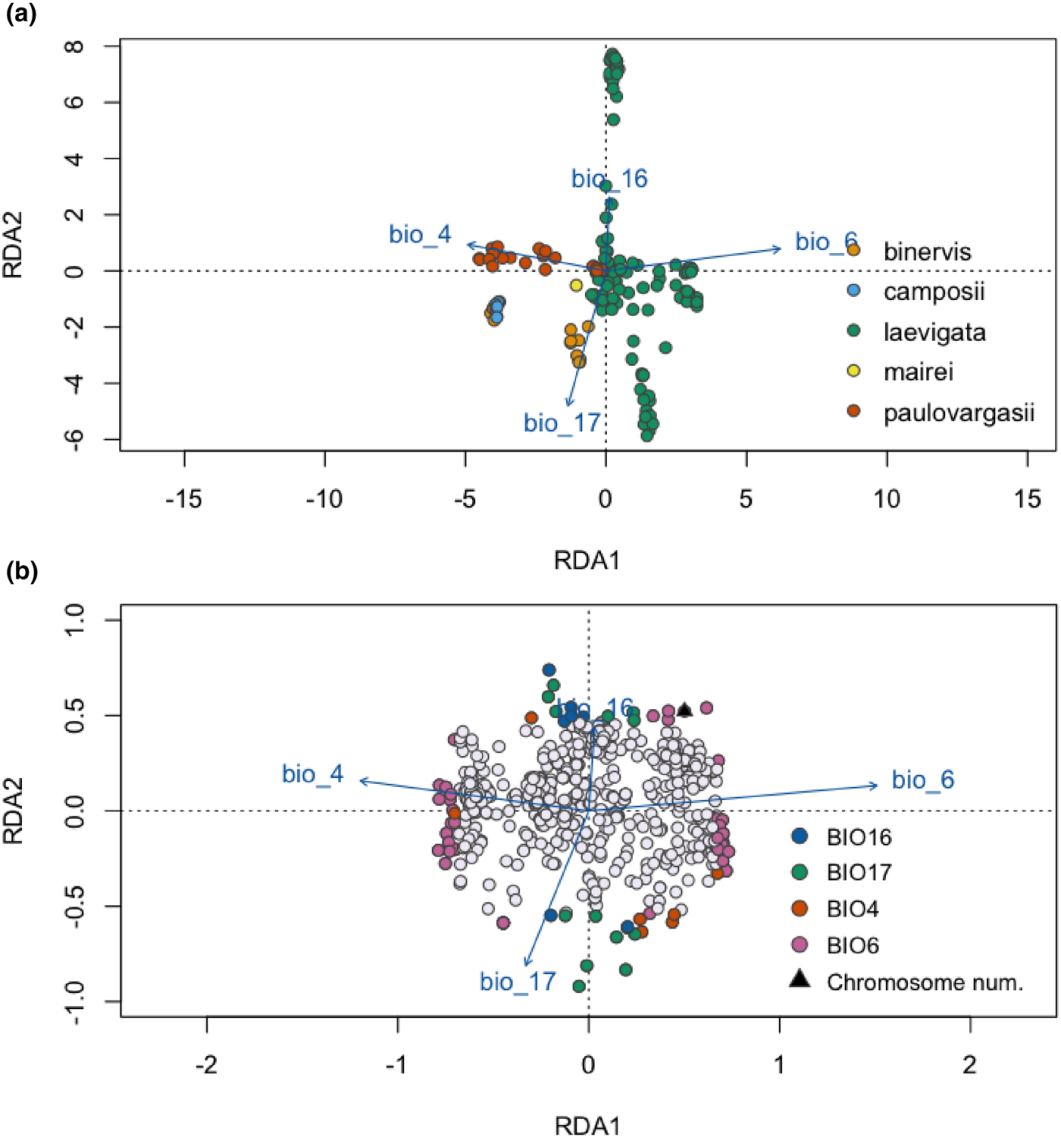
The RDA analysis. (a) Dots represent the coordinates of populations in the biplot of the first two RDA axes with colours indicating *Carex* species. (b) Dots represent the coordinates of SNPs in the biplot of the first two RDA axes and coloured dots represent outlier SNPs showing the highest correlation values with colour‐coded environmental predictors. The projection of environmental predictors used in the RDA analysis is also displayed.

Uncorrelated variables (*r* < .7) retained for the RDA analysis were BIO4 (temperature seasonality—standard deviation × 100—), BIO6 (minimum temperature of the coldest month), BIO16 (precipitation of wettest quarter) and BIO17 (precipitation of driest quarter). Permutation tests showed statistical significance for the first two axes and all four environmental variables (*p*‐value < .01). Bioclimatic variables included in the RDA explained 17.2% of the genetic variation (inertia of constrained axes), and the remaining 82.8% of the variation is unexplained and not accounted for by the included bioclimatic variables (inertia of unconstrained axes). Eigenvalues for the two significant constrained axes were 84.65 (proportion of variance explained (PVE): 0.70) for RDA1 and 19.41 (PVE: 0.16) for RDA2. RDA analysis (Figure 4a) showed a clear association between *C. paulo‐vargasii* genotypic information and BIO4, and between Iberian *C. binervis* populations and BIO17. On the other hand, Eurosiberian *C. binervis* populations, in congruence with the phylogenetic relationships, formed a distinct cluster that overlapped with *C. camposii*. Finally, *C. laevigata* populations showed a wider spread across RDA axes, although two groups of populations mostly fitted BIO16 and BIO17 values, whereas another group placed more along BIO6.

We obtained a set of 72 outlier loci putatively highly associated with the set of retained environmental predictors (Figure 4b; Table S4). Thus, BIO6, BIO17, BIO16 and BIO4 showed the strongest correlation with 43, 14, 8 and 7 outlier SNPs, respectively. Remarkably, the chromosome number of individuals used as a response variable was retrieved as an “outlier locus” in the RDA analysis and showed the strongest correlation with BIO6 (Figure 4b).

There are four loci that are significantly associated with BIO6 and highly correlated with chromosome number (Table S2). One of the four loci is linked to genome regions with high amounts of repetitive DNA. The other three loci are linked to important metabolic genes. One locus was found within a urease subunit (related to urea metabolism; although four other important genes were very close: lipoxygenase protein, aspartyl aminopeptidase, adenylate kinase and homologous to rice gene LOC). A second locus was found within a diacylglycerol kinase (DGK, related to diacylglycerol metabolism, a lipid from cell membrane; a second protein kinase domain was found very close). Finally, the last locus was quite close (ca. 10,000 bp) to a formyl transferase (Table S5).

The second analysis, excluding chromosome number as a locus, displayed identical results but without the inferred correlation between chromosome number and environmental variables (Figure S4). The third RDA analysis, with 53 individuals (only one individual pero chromosome number and per populations was kept), displayed a stronger pattern with 252 outlier loci with significant association to the climate variables (Figure S4). Chromosome number was again detected as an outlier significantly associated with BIO6, and the same four loci were tightly associated with chromosome number (Figure S4).

## 4 DISCUSSION

### 4.1 Phylogeography constrains chromosome number distribution in the western Mediterranean *Carex gr. laevigata*

Phylogeographic and phylogenetic inference in *C*. gr. *laevigata* has remained obscure in previous studies based on Sanger sequencing of a few DNA regions, especially for the *C. laevigata*‐*C. binervis* species pair (Escudero et al., 2008, 2010; Escudero & Luceño, 2009). On one hand, systematic studies focused on the *Carex* sect. *Spirostachyae* based on ITS and 5’*trn*K intron (Escudero et al., 2008, 2010; Escudero & Luceño, 2009) retrieved non‐monophyly and intermingled phylogenetic relationships for *C. laevigata*‐*C. binervis*. On the other hand, the only previous attempt to elucidate the phylogeographic structure of the group, based on two plastid DNA regions (5'*trn*K and the intergenic *trn*V‐*ndh*C) and a wide population sampling, obtained a highly intricate haplotype network (Escudero, Maguilla, et al., 2013). Previously inferred ribotype additivities and haplotype sharing between *C. laevigata*‐*C. binervis* have been attributed to hybridisation (Escudero et al., 2008; Escudero, Maguilla, et al., 2013; Escudero & Luceño, 2009) or incomplete lineage sorting (Escudero, Maguilla, et al., 2013). The phylogenetic resolution provided by the highly increased amount of loci obtained across the entire genomes of the study species with the RAD‐seq approach (1761 loci here vs. 1–2 in previous studies) allowed us to shed light on the main phylogeographic patterns of *C*. gr. *laevigata*. While the phylogenetic relationships for *C. camposii* and *C. paulo‐vargasii* are congruent with previous studies, we uncovered a previously unknown genetic lineage for *C. binervis* from the Eurosiberian part of its range, and distinct from the clade including *C. binervis* samples from the Iberian Peninsula. Overall, the southern Iberian peninsula‐north Africa displayed higher phylogenetic, genetic, taxonomic (all four species of *C*. gr. *laevigata* present) and cytogenetic (most chromosome number range) diversities (Figures 1, 2, 3, 4). Besides, endemics from that region (*C. paulo‐vargasii* from Morocco and *C. camposii* from Sierra Nevada), as well as southern populations of *C. binervis* and *C. laevigata* appeared as successive sisters to the rest of the lineages (Figure 2). On the other hand, only *C. laevigata* and *C. binervis* are distributed in the northern Iberian Peninsula and the rest of western Europe, where a reduced subset of their phylogenetic diversity (Figure 2) and chromosome numbers are found (2*n* = 70–74, Figure 1). Therefore, the taxonomic, molecular and cytogenetic evidence points to southern Iberia‐north Africa (especially the region around the Strait of Gibraltar) as the evolutionary cradle for *C*. gr. *laevigata*. These phylogeographic and karyotypic patterns are compatible with the signature of Pleistocene glaciations, including southern refugia and northward postglacial recolonisation (e.g. Hewitt, 2011). This temporal‐geographic scenario (Pleistocene diversification—Martín‐Bravo et al., 2019—within a large latitudinal gradient in southwestern parts of the Western Palaearctic), which frequently caused speciation (e.g. Kadereit & Abbott, 2021), has been previously reviewed for many plant groups (Lavergne et al., 2013; Molina‐Venegas et al., 2017; Nieto Feliner, 2014; Rodríguez‐Sánchez et al., 2008).

### 4.2 Landscape genomics meets karyotype evolution

Despite alleged limitations of RAD‐seq genomic scanning to detect loci related to local adaptation (Lowry et al., 2017), our landscape genomic analyses have shown that 74 outlier loci are significantly correlated with climatic variables related to temperature (BIO4 and BIO6) and precipitation (BIO16 and BIO17). Interestingly, chromosome number was among these outliers and was also correlated with BIO6. Together with chromosome number we also identified four loci significantly correlated with BIO6 and with similar associations with RDA1 and RDA2 axes as chromosome number (Figure 3b).

One of the four loci is linked to genome regions with high amounts of repetitive DNA. This is coherent as highly repetitive sequences can be found at centromeres and telomeres or in holocentromeres in holocentric chromosomes (Hofstatter et al., 2022), which play critical roles in genome integrity maintenance throughout the cell cycle (Onishi‐Seebacher & Korbel, 2011). Repetitive DNA sequences were previously referred to as “junk DNA” because few discernible functions could be assigned to these regions, but recent studies have shown that they have important functional roles in genome organisation and evolution (Biscotti et al., 2015; von Sternberg & Shapiro, 2005). Repetitive DNA has been associated with ectopic recombination (González & Petrov, 2012), also in holocentric organisms (Höök et al., 2023), which could result in chromosomal fission and fusion rearrangements and explain, at least partially, the resulting patterns of karyotype variation. In this way, very recently, the genomic architecture of fission and fusion has been deciphered for holocentric sedges (Escudero, Marques, et al., 2023; Hofstatter et al., 2022) and butterflies (Höök et al., 2023), which is characterised by a high density of repetitive DNA.

The other three significantly correlated loci were associated with genes involved in important metabolic routes. Kinases were especially relevant (three hits) and associated to two of the three loci (Table S5). Among these, diacylglycerol kinase (DGK) genes are of special relevance given that they are involved in the modulation of plant growth, development, and adaptation in both biotic and abiotic stress conditions (Kue Foka et al., 2020). Additionally, DGK plays an important role in generating membrane‐derived oligosaccharides that protect cells against osmotic stress conditions (Jefferson et al., 2013). Furthermore, glycerophospholipids (GPL), which are synthesised by DGK, have been linked with adaptation to high‐altitude cold environments (Wei et al., 2022). Kinase domains seem to be associated with stress response and adaptation to the environment in plants. Receptor‐like kinases (RLKs) and histidine kinases have been found to play a role in plant response to abiotic stresses (Osakabe et al., 2013). Additionally, calcium‐dependent protein kinases (CDPKs) are essential for plant development and stress responses (Alves et al., 2021). SNF1‐related protein kinases have been found to have a unique function in plant glucose and stress signalling (Lumbreras et al., 2001). Furthermore, receptor‐like kinases have been found to play significant roles in plant growth regulation and responses to stresses (Fenglian et al., 2012). Finally, kinase domains have also been found to function in plant growth and salt‐stress responses (Zhou et al., 2006).

Remarkably, different chromosome numbers (which are related to linkage groups and recombination rates) are significantly associated with allelic variants at these loci (with important genes), which in turn are also significantly associated with environmental climatic variables. This study is a step forward in supporting the hypothesis that cytogenetic variation (chromosome number, linkage groups and recombination rates) in species with holocentric chromosomes are selected toward different optima of climatic regimes (Escudero et al., 2012; Escudero, Maguilla, et al., 2013; Márquez‐Corro et al., 2021; Spalink et al., 2018).

### 4.3 Implications for speciation

Karyotype diversity is directly associated with diversification rates and species richness in angiosperms (Carta & Escudero, 2023), which suggests a key role of chromosome evolution in the plant diversification process. Chromosome evolution has been hypothesised as a major driver of diversification in the Mediterranean Basin (Escudero et al., 2018; Thompson, 2020). In addition, chromosome evolution has been suggested to play a major role in species diversification in holocentrics (de Vos et al., 2020; Escudero et al., 2010; Escudero, Maguilla, et al., 2013; Hipp et al., 2010). Finally, chromosome evolution may also have an indirect role reinforcing geographical or ecological speciation (Coyne & Orr, 2004).

There are two overarching models for chromosomal speciation, hybrid dysfunction and recombination suppression (Ayala & Coluzzi, 2005). They are not mutually exclusive and they can both play a partial role in the process of chromosomal speciation, especially in holocentric chromosomes where hybrid dysfunction is better supported theoretically than in monocentrics (Lucek et al., 2022). The suggested adaptive chromosomal speciation here could be helpful to support both models of chromosomal speciation. On one hand, strong selection toward new chromosomal variants may help them to become established in the populations which can ameliorate one of the strongest criticisms against the hybrid dysfunction model (the minority cytotype exclusion; Levin, 1975). Our genetic structure analyses (Figures 2 and 3) display how genetically very closely related individuals may have different chromosome numbers which suggest that chromosome changes by fission and fusion do not entail an instantaneous gene flow disruption and the accumulation of genetic differences (see also Escudero, Arroyo, et al., 2023). On the other hand, recombination suppression models already suggest locally adapted genes in which recombination is protected by chromosome rearrangements (Ayala & Coluzzi, 2005). Accordingly, under this chromosomal speciation model, we certainly expect that different karyotypes are locally adapted. Our landscape genomics analyses suggest that karyotypes are locally adapted in our study group. In addition, the phylogenetic placement and genetic structure of sampled populations suggests multiple origins of different chromosome numbers (e.g. 2*n* = 72, 76), which is congruent with recurrent chromosome fission and fusion processes in relatively short time periods (Escudero, Arroyo, et al., 2023) and further support its adaptive value. We hypothesise here that clusters of locally adapted genes in *C*. gr. *laevigata* might have been protected from recombination by the fission and fusion rearrangements. In fact, fissions and fusions have been observed to suppress recombination in holocentrics in a study of comparative genomics (whole sequence genome vs. linkage map, Escudero, Marques, et al., 2023). For instance, in *C. laevigata*, this would have allowed different karyotypes (and, consequently, gene combinations) to either disperse northward after the last glacial period or remain in the progressively warmer habitats in the southern Iberian Peninsula, further contributing to lineage divergence.

## AUTHOR CONTRIBUTIONS

Marcial Escudero, Santiago Martín‐Bravo and Modesto Luceño designed the research. José Ignacio Márquez‐Corro, Marcial Escudero, Santiago Martín‐Bravo and Modesto Luceño collected plant samples. Marcial Escudero and José Ignacio Márquez‐Corro prepared and sent the DNA samples to Floragenex. José Ignacio Márquez‐Corro, Marcial Escudero, José Luis Blanco‐Pastor analysed the data. José Ignacio Márquez‐Corro, Marcial Escudero and Santiago Martín‐Bravo wrote the manuscript. Modesto Luceño and José Luis Blanco‐Pastor contributed to the manuscript writing.

## CONFLICT OF INTEREST STATEMENT

The authors have no conflicts of interest to declare.

## Supporting information


Figure S1.

Figure S2.

Figure S3.

Figure S4.

Table S1.

Table S2.

Table S3.

Table S4.

Table S5.

Supinfo S1

## Data Availability

Obtained RAD‐seq data are available on the NCBI under BioProject accession PRJNA982410.
