# Supplementary figures and images for: The holocentric chromosome microevolution: From phylogeographic patterns to genomic associations with environmental gradients
Source: Mol Ecol. 2023 Oct 5;33(24):e17156. doi: 10.1111/mec.17156 (PMC11628669; doi:10.1111/mec.17156)

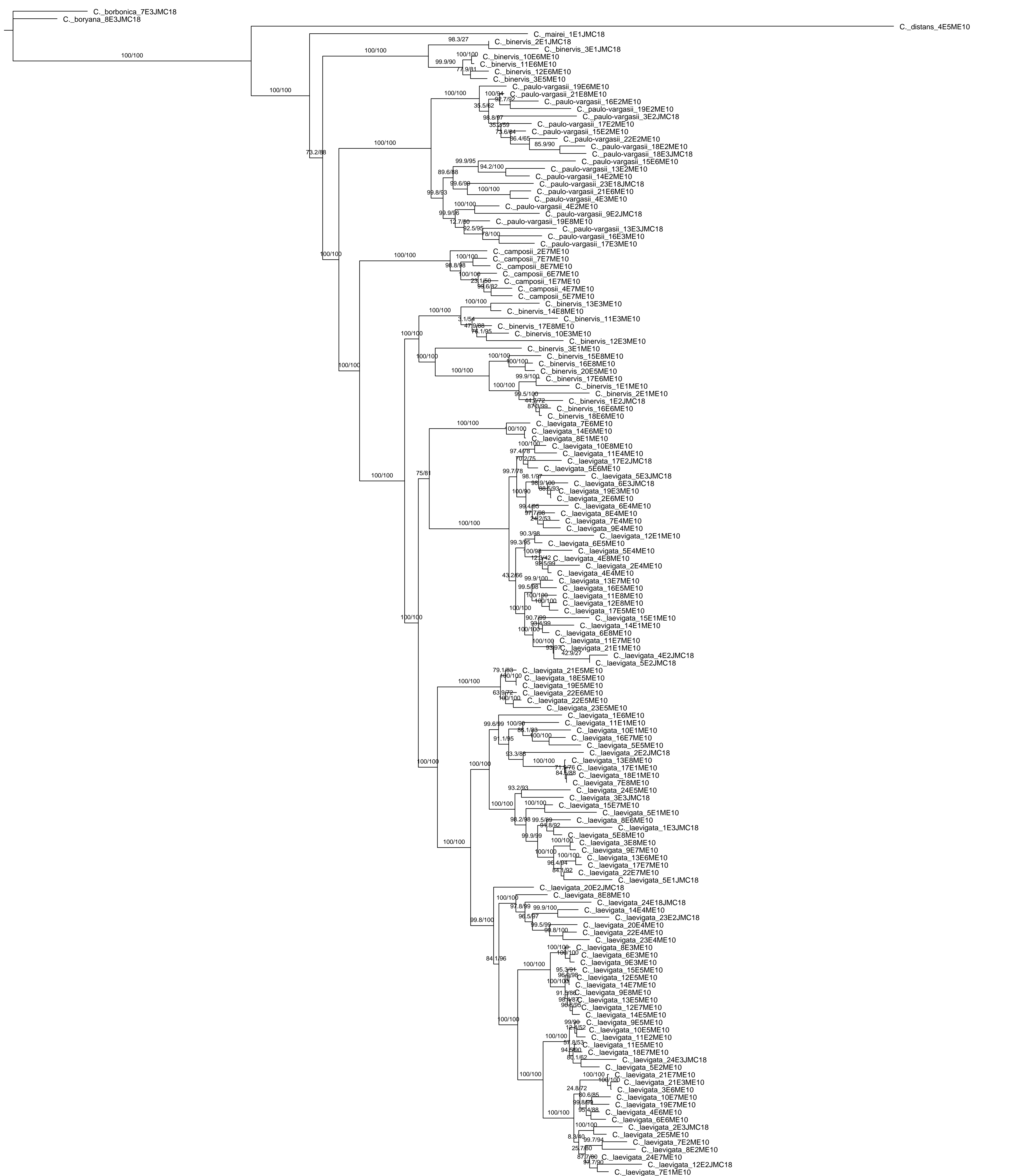

Supplement: Supplementary file 1 — Figure S1. [file MEC-33-e17156-s004.pdf]

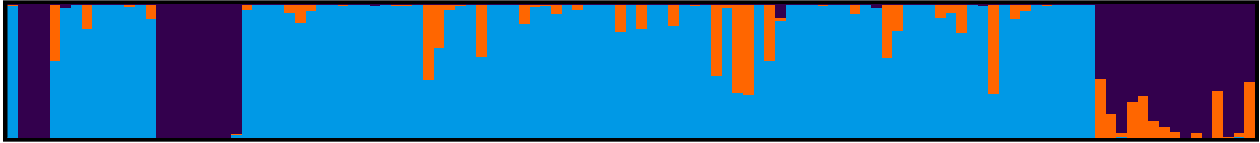

Supplement: Supplementary file 2 — Figure S2. [file MEC-33-e17156-s008.pdf]

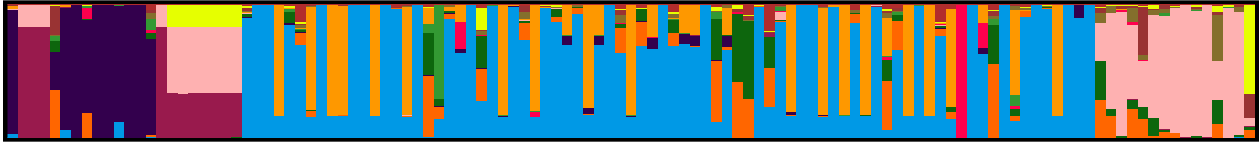

Supplement: Supplementary file 3 — Figure S3. [file MEC-33-e17156-s010.pdf]

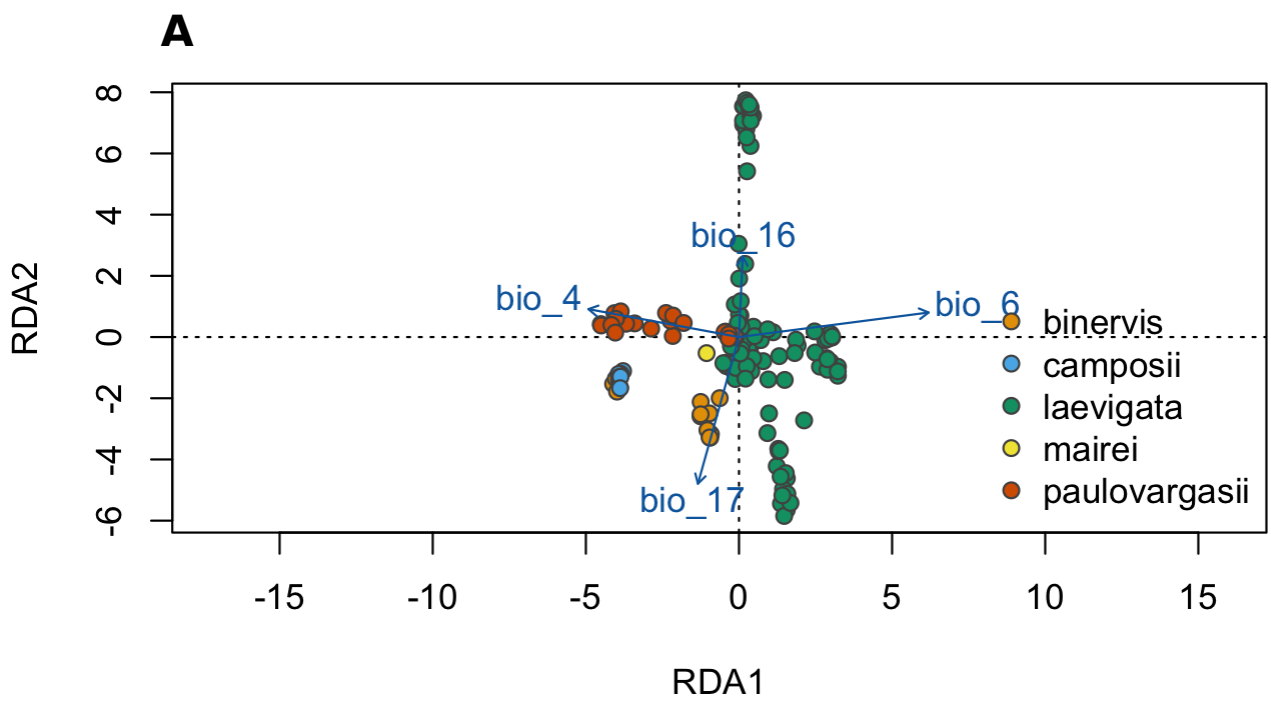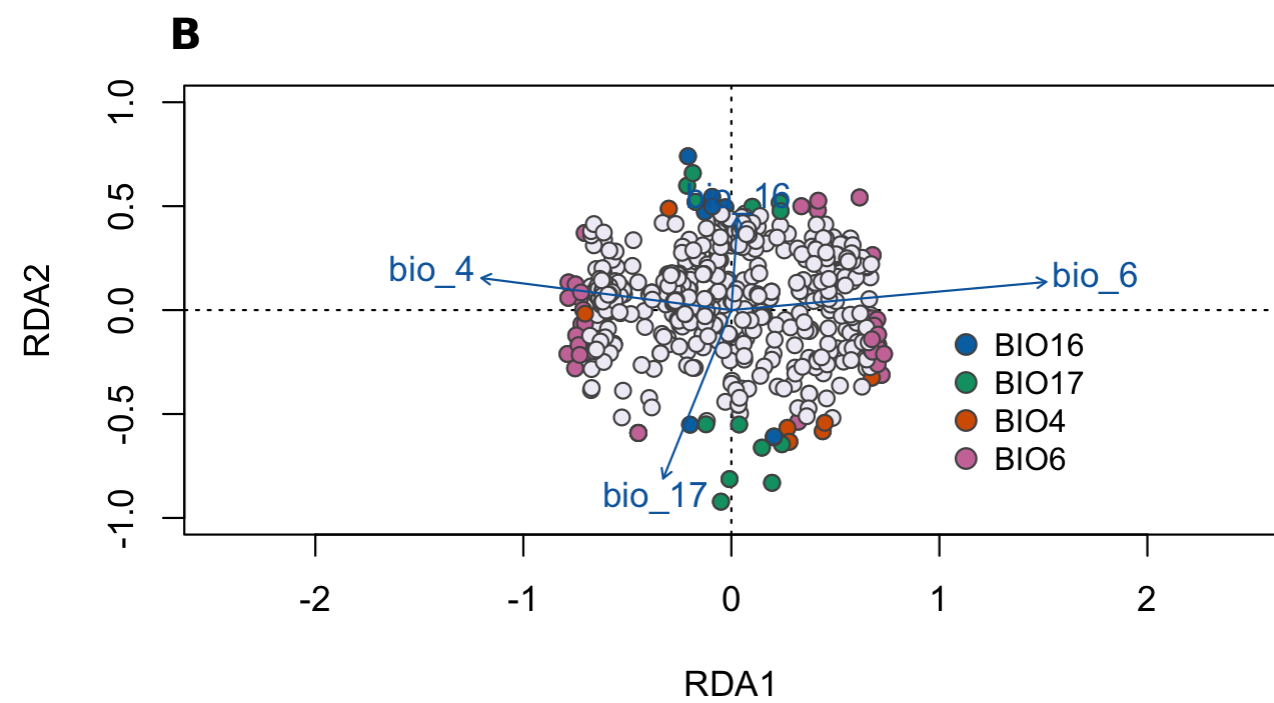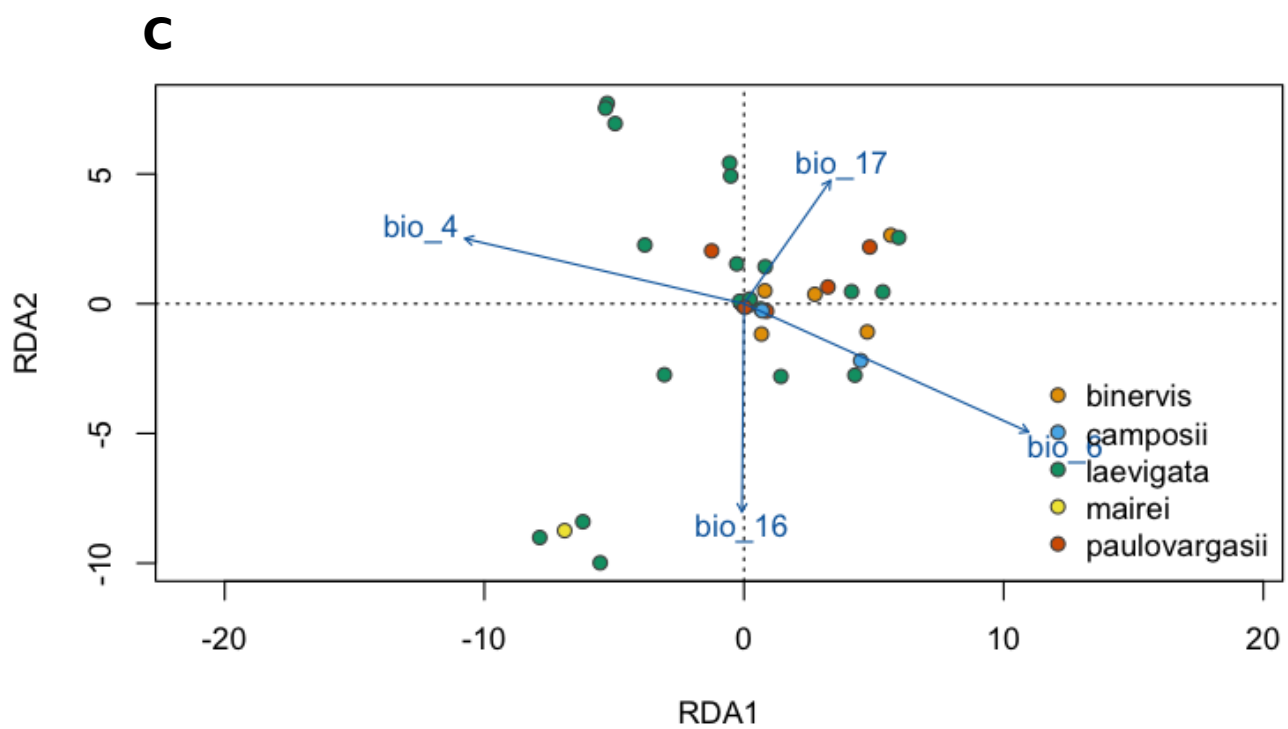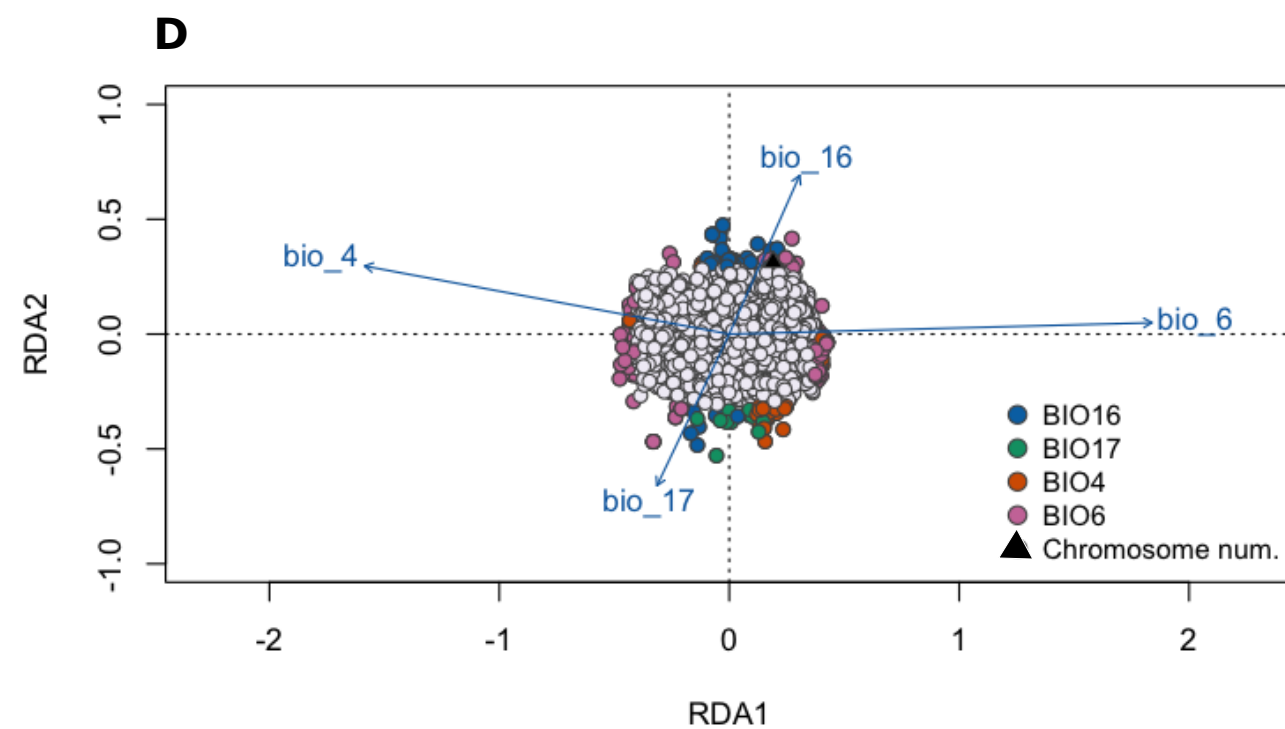

Supplement: Supplementary file 4 — Figure S4. [file MEC-33-e17156-s006.pdf]
