# Supplementary material for: The holocentric chromosome microevolution: From phylogeographic patterns to genomic associations with environmental gradients
Source: Mol Ecol. 2023 Oct 5;33(24):e17156. doi: 10.1111/mec.17156 (PMC11628669; doi:10.1111/mec.17156)
Supplement: Supplementary file 10 — Supinfo S1 [file MEC-33-e17156-s001.docx]

Figure S1. RAD-seq phylogeny of 156 samples of *Carex* gr. *laevigata* plus the outgroup.

Figure S2. STRUCTURE analysis of *Carex* gr. *laevigata*. Results of the clustering for K = 13.

Figure S3. STRUCTURE analysis of *Carex* gr. *laevigata*. Results of the clustering for K = 3.

Figure S4. The RDA analyses excluding chromosome number as locus (A and B) and including only one individual per population and chromosome number (C and D). (A and C) Dots represent the coordinates of populations in the biplot of the first two RDA axes with colours indicating Carex species. (B and D) Dots represent the coordinates of SNPs in the biplot of the first two RDA axes and coloured dots represent outlier SNPs showing the highest correlation values with colour-coded environmental predictors. The projection of environmental predictors used in the RDA analysis is also displayed.

Table S1. Sampling information. For each sample SRA accession, specimen code (species, population and individual number), voucher information, chromosome data (karyotype and 2*n* number), DNA extraction code and passing of RADseq quality filters are provided for all sampled material (included outgroup) used in the study.

Table S2. Population summary. Initial number of specimen sampled is depicted together with the retained numbers after each filter is applied for the different analysis.

Table S3. STRUCTURE output from K = 1 to K = 30 values. The two best clustering results (K = 2 and K = 6) are shown in bold.

Table S4. The 72 outlier loci associated with environmental variables from RDA analysis 1.

Table S5. The four loci associated with chromosome number and BIO6 are shown indicating locus number, chromosome location in *C. scoparia* reference genome, location in base pairs (bp), the closest genome feature and its location and other genomic features found within 50 Kbp range upward and downward.
